# Collectanea Medica, Consisting of Anecdotes, Facts, Extracts, Illustrations, Queries, Suggestions, &c.

**Published:** 1817-06

**Authors:** 


### COLLECTANEA MEDICA,

CONSISTING OF

### ANECDOTES, FACTS, EXTRACTS, ILLUSTRATIONS, QUERIES, SUGGESTIONS, &c.

RELATING TO THE

*History or the Art of Medicine, and the Auxiliary Sciences.*

Quicquid agunt medici, nostri farrago libelli.

### *Extract from a Journey through Egypt;* by Thomas Legh, Esq. M. P.

THE following interesting account of the mephitic vapour of a cavern is taken from the above work.

"We were bent on going, and the Arabs at last undertook to be our guides for a reward of twenty-five piastres. After an hour's march in the desert, we arrived at the spot, which we found to be a pit or circular hole of ten feet in diameter, and about eighteen feet deep. We descended without difficulty, and the Arabs began to to strip, and proposed to us to do the same: we partly followed their example, but kept on our trowsers and shirts. I had by me a brace of pocket pistols, which I concealed in my trowsers, to be prepared against any treacherous attempt of our guides. It was now decided that three of the four Arabs should go with us, while the other remained on the outside of the cavern. The Abyssinian merchant declined going any farther. The sailors remained also on the outside to take care of our clothes. We formed, therefore, a party of six; each was to be preceded by a guide—our torches were lighted—one of the Arabs led the way,—and I followed him.

"We crept for seven or eight yards through an opening at the bottom of the pit, which was partly choaked up with the drifted sand of the desert, and found ourselves in a large chamber about fifteen feet high.

"This was probably the place into which the Greek, Demetrius, had penetrated; and here we observed what he had described, the fragments of the mummies of crocodiles. We saw also great numbers of bats flying about, and hanging from the roof of the chamber. Whilst holding up my torch to examine the vault, I accidentally scorched one of them. I mention this trivial circumstance, because afterwards it gave occasion to a most ridiculous, though to us very important, discussion.* So far the story of the Greek was true, and it remained only to explore the galleries where the Arabs had formerly taken refuge, and where, without doubt, were deposited the mummies we were searching for. We had all of us torches, and our guides insisted upon our placing ourselves in such a way, that an Arab was before each of us. Though there appeared something mysterious in this order of march, we did not dispute with them, but proceeded. We now entered a low gallery, in which we continued for more than an hour, stooping or creeping as was necessary, and following its windings, till at last it opened into a large chamber, which, after some time, we recognized as the one we had first entered, and from which we had set out. Our conductors, however, denied that it was the same, but on our persisting in the assertion, agreed at last that it was, and confessed they had missed their way the first time, but if we would make another attempt they would undertake to conduct us to the mummies. Our curiosity was still unsatisfied; we had been wandering for more than an hour in low subterranean passages, and felt considerably fatigued by the irksomeness of the posture in which we had been obliged to move, and the heat of our torches in those narrow and low galleries. But the Arabs spoke so confidently of succeeding in this second trial, that we were induced once more to attend them. We found the opening of the chamber which we now approached guarded

* They were accused by the natives of raising fire by Magic.

by a trench of unknown depth, and wide enough to require a good leap. The first Arab jumped the ditch, and we all followed him. The passage we entered was extremely small, and so low in some places as to oblige us to crawl flat on the ground, and almost always on our hands and knees. The intricacies of its windings resembled a labyrinth, and it terminated at length in a chamber much smaller than that which we had left, but, like it, containing nothing to satisfy our curiosity. Our search hitherto had been fruitless, but the mummies might not be far distant; another effort, and we might still be successful.

"The Arab whom I followed, and who led the way, now entered another gallery, and we all continued to move in the same manner as before, each preceded by a guide. We had not gone far before the heat became excessive;—for my own part I found my breathing extremely difficult, my head began to ache most violently, and I had a most distressing sensation of fulness about the heart.

"We felt we had gone too far, and yet were almost deprived of the power of returning. At this moment the torch of the first Arab went out: I was close to him, and saw him fall on his side; he uttered a groan—his legs were strongly convulsed, and I heard a rattling noise in his throat—he was dead. The Arab behind me, seeing the torch of his companion extinguished, and conceiving he had stumbled, past me, advanced to his assistance, and stooped. I observed him appear faint, totter, and fall in a moment—he also was dead. The third Arab came forward, and made an effort to approach the bodies, but stopped short. We looked at each other in silent horror. The danger increased every instant; our torches burnt faintly; our breathing became more difficult; our knees tottered under us, and we felt our strength nearly gone.

"There was no time to be lost—the American, Barthow, cried to us to 'take courage;' and we began to move back as fast as we could. We heard the remaining Arab shouting after us, calling us Caffres, imploring our assistance, and upbraiding us with deserting him. But we were obliged to leave him to his fate, expecting every moment to share it with him. The windings of the passages through which we had come increased the difficulty of our escape; we might take a wrong turn, and never reach the great chamber we had first entered. Even supposing we took the shortest road, it was but too probable our strength would fail us before we arrived. We had each of us separately, and unknown to one another, observed attentively the different shapes of the stones which projected into the galleries we had passed, so that each had an imperfect clue to the labyrinth we had now to retrace. We compared notes, and only on one occasion had a dispute, the American differing from my friend and myself: in this dilemma we were determined by the majority, and fortunately were right. Exhausted with fatigue and terror, we reached the edge of the deep deep trench which remained to be crossed before we got into the great chamber. Mustering all my strength, I leaped, and was followed by the American. Smelt stood on the brink, ready to drop with fatigue. He called to us 'for God's sake to help him over the fosse, or at least to stop, if only for five minutes, to allow him time to recover his strength.' It was impossible—to stay was death; and we could not resist the desire to push on and reach the open air. We encouraged him to summon all his force, and he cleared the trench. When we reached the open air it was one o'clock, and the heat in the sun about 160°. Our sailors, who were waiting for us, had luckily a *bardak** full of water, which they sprinkled upon us; but, though a little refreshed, it was not possible to climb the sides of the pit: they unfolded their turbans, and, slinging them round our bodies, drew us to the top."

*Miscellaneous Hints concerning the Plague* (from the same).

"There is something inexplicable, and that one might be disposed to call capricious, in the way in which the plague spreads itself from one country to another; and we had been particularly struck with the observation of the Greek who acted as English Consul at Scio. Though within a few hours sail of Smyrna, where numbers were dying daily of the plague, he had no fear of its approaching the island; and, during our stay of some days, we saw many Turks, who had come from that place, leap on shore without any interruption. But, added the Consul, should the plague declare itself at Alexandria, distant some hundred miles, we shall certainly have it at Scio. He spoke confidently, and quoted many instances within his own memory of the like coincidence.

"To our great disappointment, however, we found, on our arrival at Old Cairo, that the plague had declared itself in that city; that all the Franks had shut themselves up; and that even the Pacha had removed to Gizeh, with which place he would allow no communication. So strict were his orders, that any boat attempting to pass on the western side of the Nile, and consequently near his residence, was fired upon. The other side of the river was so shallow as not to be navigable; there was, therefore, at this point a complete interruption of all intercourse between the Delta and Upper Egypt. The Pacha had also established a quarantine of ten days at Rosetta, and as this is the first instance with which I am acquainted of the use of precautions against the plague by a Turkish authority, it may be considered an important step towards civilization. It was from early habits that the Pacha had become familiar with the customs of European policy, and his active and enterprizing mind adopted its improvements without any

"* The name of the jars, made at Kenne, of porous earth, and used to cool water."

any regard to the prejudices and superstitions of his own Mahometan faith.*"

The following is the history of the author's residence during the plague at Rosetta, and of the process on his arrival in England.

" The house we occupied had double doors, and in the space between them we placed two very large jars filled with water, which was changed once in the 24 hours; and having provided ourselves also with a fumigating box, to receive all our letters, we hired an Arab for a piastre a day, to station himself every morning under our windows, receive our orders, and purchase our provisions.

" With respect to our bread, we took the precaution of never touching it till it was cool, as it is ascertained that in that state it does not communicate the plague. Even letters which have been fumigated must be allowed to cool before they are touched.

" Our meat, whether beef or fowls, the latter being previously plucked, was all thrown into the water jars, from which, after a certain interval, it was cautiously taken out by one of our servants, who opened the inner door for the purpose. In this manner we lived for several weeks, witnessing the most distressing sights of death and disease under our windows, from which we had frequent opportunities of observing attacks of the plague, as it first seized upon its unfortunate victims. As far as we could judge from their gestures, they appeared to suffer most violent pains in the head, and were at the same time seized with violent retchings, and black vomiting.

" We lost three of the Arabs, whom we had engaged to act as our purveyors in the town. When the mortality was at its height, the numbers who died daily amounted to about eighty.

" It was impossible, however, to include in our measures of safety the few English soldiers who were employed, together with about 50 Arabs, in looking after the horses piqueted in the camp without the town; but the judicious directions of their officers, and the ready obedience of the men in avoiding every occasion of touching either the native servants, several of whom died, or the horses of which they had the immediate care, saved them from any infection.

" The exemption of the British soldiers from the attacks of the disease is an additional instance in support of the opinion that the plague is only to be communicated by actual contact; for they were exposed to the same atmosphere, and to the action of the same

---

" * Owing to the measures adopted by the Pacha, the plague, which shewed itself in February at Alexandria, did not make its appearance in Cairo before the commencement of the hot weather. So much of its violence was abated at this period, that the greatest mortality we heard of in that city, where the population is estimated at nearly 400,000, did not exceed fifty a-day."

same general causes, as the less fortunate natives who, like themselves, were employed in the care of the horses.

"At one time, more than 2000 of the population of Rosetta were said to be ill of the plague, of whom the greatest number did not confine themseves to their houses, but were seen walking about, to the great danger of the rest of the inhabitants. The Arabs and Turks, having no fear of the contagion, are in consequence always ready to lend every assistance to their sick friends; and it is perhaps partly to be attributed to this cause that a greater number of Mahometans recover than Europeans, the latter being generally deserted by their countrymen. The fearlessness of danger and humane attentions of the natives occasion, however, a great spreading of the contagion, to which the custom that prevails amongst them, for the nearest relation to wear the clothes of the deceased, in the last duties paid to his memory, does not a little contribute.

"We heard of no remedy for the plague: when the swellings broke, sea bathing was supposed to be very beneficial, but after that event the patients generally recovered without any remedy.

"We found the opinion that the disease ceases on the 24th June, St. John's day, prevalent among the Franks as well as the natives of the country.

"The Europeans settled at Cairo and Alexandria would not open their houses before that time, when they began to have cautious communication with their neighbours. This period, however, which had been so eagerly anticipated, and whose approach was hailed by the lighting of several bonfires in different parts of the town, did not, on this occasion, answer the general expectation, but on the contrary was marked by an unusual mortality, as the deaths on that day exceeded a hundred, a number considerably beyond the usual average.

"Much beneficial effect is also attributed to the *Nokta*, or rising of the Nile, which begins on the 18th June. Previous to this month the Kamsin, or Wind of the Desert, which commences generally on Easter Monday, and continues to blow for fifty days, together with the stagnant state of the waters of the Nile, are supposed to occasion the unhealthiness generally observed to prevail at that season. So confirmed is this idea, that the Arabs are in the habit of congratulating one another at the end of the Kamsin, on having escaped its baneful effects.

"The two or three months previous to the summer solstice are reputed so unhealthy, that the plague is said to exist in Cairo always during that period, at which time also the small-pox is very fatal.

"When the natives are seized with the first symptoms of the plague, they wrap themselves up in their cloaks, and endeavour to promote perspiration by drinking large quantities of warm water. In a short time, swellings break out in the groin and under the arms; and if they are alive thirty-six hours after the first seizure, they generally recover. We saw a Turk at Alexandria who who had suffered several attacks of the plague, and he informed us, that, as soon as he was able to move, he crawled to the sea side, in which he constantly bathed.

"Neither iron nor wood convey the infection, though money is supposed to do so, a circumstance, perhaps, to be attributed to the custom that prevails amongst the natives of carrying it in a small bag worn close to the skin. In this situation it is certainly more likely to imbibe the matter of contagion secreted on the surface of the body; but, whatever may be the cause, we always took the precaution to allow our money to remain in the water at least half an hour before we touched it.

"Such was the plan of life we adopted; and the success of our measures of precaution abundantly proves the utility and sufficiency of the usual quarantine regulations established in the countries of the Mediterranean, which are frequently visited by the calamities of the plague. But, on our return to England, it was impossible not to smile at the insufficiency, not to say absurdity, of the system adopted in this country. As we passed up the Channel, we were visited by the officers of the Board of Health; and one of them coming alongside our vessel, presented the Captain with a Bible, requesting him to swear to the truth of the answers he should make to his several questions. It was in vain we represented to him, that his taking the book again from our hands would be the surest means of communicating to him whatever infection we might ourselves be labouring under: he persisted in demanding our compliance with a form which could not be dispensed with, and added, with an air of triumph, that, in the discharge of his duty, he had himself been on board several plague ships, with impunity. On the same occasion, another officer produced a number of queries, to which the captain of our vessel was required to give written answers; and when told nothing was so infectious as paper, he contented himself with replying, that the orders of the Privy Council were peremptory, and must be obeyed."

We shall, at present, only express our surprise that an author, who gives a native Greek credit for such accuracy in tracing what he is disposed to call "inexplicably capricious in the way in which the plague spreads," should be so confident in admitting these various opinions concerning contact, hot and cold bread, &c. all of which we conceive the sagacious, or, at least, experienced Greek, would have smiled at as an English prejudice. That the Arabs died, cannot be a matter of surprise, when we consider that their occupation led them to every part of the town; and, perhaps, it will be found that the British soldiers escaped, not by avoiding contact with the horses, but because they were confined to a district without the town. As Mr. Legh is not a professional gentleman, we have treated his opinions with the less reserve; but it is truly surprising that no medical traveller has hitherto attempted to explain the cause of the various contradictory opinions on this important question.

It

It may not be amiss, on this occasion, to add a few paragraphs from Dr. Russell's valuable Treatise on the Plague. By these we shall find that this industrious, honest, and we may add, for the most part, candid author is equally puzzled at "something inexplicably capricious in the manner in which the plague spreads;" yet equally backward in attending to the suggestions of experience which he might have derived from the best informed among the natives.

*Account of the Plague in Cyprus*, (from Dr. Russell.)

"The plague was brought to Cyprus in the month of April, 1759, and, its progress in that island having been attended by some remarkable circumstances, I shall here give the following extract from an account that now lies before me; on the fidelity and accuracy of which, I can venture to depend.

"In the month of April, 1759, a large Turkish vessel, laden at Alexandria, and bound for Constantinople, was wrecked in her passage, not far from Cape Baffo. Of the crew who were saved, a great part happened to be infected with the plague; which was first communicated to certain villages on the road to Limsol, and afterwards to that town itself.

"Some of the sailors died in the villages. The rest, after a short stay at Limsol, proceeded to Larnica; where they remained only a few days, till a vessel presented, in which they crossed over to Syria. None of them died at Larnica; though it was known that several actually had the plague.

"The contagion spread with such rapidity at Limsol, that, in the month of June, upwards of four hundred persons were reckoned to have died of it. Many of the inhabitants fled to the neighbouring villages, and to the mountains, transporting the contagion along with them. But, though the plague showed itself, now and then, in those parts where the fugitives had taken shelter, as well as in other inland villages which had intercourse with Limsol; yet it was only about Baffo, and near to Limsol, that it spread considerably.

"The condition of Larnica, at this period, was remarkable. It had received part of the infected crew from Limsol; it had maintained a constant intercourse with the infected quarters of the island; peasants and mule-drivers from those parts, with the pestilential sores on their bodies, were daily in the streets and markets; and some of them died in the houses of Larnica. On the 22d of May, a vessel arrived from Damietta, which put on shore some infected passengers and sailors, who lodged in the houses, and communicated freely with the natives. Another Turkish vessel, from the same place, arrived, some time after, with infected persons on board; one of which died on landing at the Marine. Notwithstanding this new importation, none of the inhabitants of Larnica were known to have contracted the plague. The Europeans, from whom many of the above circumstances were, at the time, carefully kept secret, observed no precautions for for their own safety; while the natives consoled themselves with a traditionary notion, that a plague which did not begin in December was not to be dreaded.

"During the hot months of July, August, and September, little was heard of the plague; and it was generally supposed to be extinguished at Limsol as well as in the villages; but, the truth was, it had all along continued lurking in those parts, showing itself only by starts; particularly at Baffo, Piscopi, and other villages on the western and southern sides of the island.

"In the month of October, the plague increased in those parts, where it had appeared in the spring, and, soon after, broke out at Nicosia; to which place, the annual fair of St. Demetrio had drawn a great concourse of people from most parts of the island. The magistrates of Nicosia endeavoured, at first, to conceal the nature of the distemper, under the name of a malignant fever; and, in December, when eight or ten died daily, the dead bodies were buried privately in the night, in order to prevent the inhabitants being alarmed by frequent funerals. But, towards the end of the year, matters became too serious for stratagems of this kind; for the contagion, which had, for some time before, got among the Greeks and Armenians, was now arrived at such a height, as, on some days, to carry off fifteen Christians, which people, in number, bear a small proportion to the Mohammedans.

"In the beginning of February, the distemper appeared among the Turks at the Marine; and, soon after, at Larnica. The Europeans shut up. The daily burials soon increased to eight or ten; and, during February, never exceeded twenty. In the month of March, the disease would appear to have been more malignant than at first; for few or none of the infected recovered. The daily funerals arose to twenty-five and thirty; and many of the inhabitants fled to the mountains.

"The distemper continued to rage at Larnica all the month of April, and spread, at the same time, over the island, penetrating even into the province of Carpass, a circumstance not known to have ever happened before. At the Marine, the daily burials decreased, which was attributed to the flight of the wretched inhabitants, many of whom, like those of Larnica, abandoned their half desolate houses, and sought refuge in the country.

"In this month also died several of the Europeans at Larnica. In the family of the Neapolitan Consul, a French gentleman, who lodged in the house; two children; the Consul himself, and several servants; all died successively. The widow of a Neapolitan, who had lost her husband in the plague some time before, gave constant attendance on the Consul, as well as the others infected of that family, without any bad consequence. M. Lefebure, a French surgeon, who had been established many years at Cyprus, died about this time. He had caught the infection in the course of his attendance on a sick person; but could not be persuaded that he himself had got the plague, 'till a few hours before his death, when the buboes appeared. This gentleman was carefully attended by his partner, by a priest, and by two servants; of whom the priest only was infected, and he died within a few days after. The above instances may serve as an answer to an assertion, boldly hazarded and often repeated, that Europeans, in Turkey, are insusceptible of the plague.

"In the month of May, died the son-in-law of the Neapolitan Consul, and some other Europeans; among whom was the Superior of the Terra Santa Convent. As this Convent had shut up at the same time with the European merchants, and was supposed to have rigorously observed all the usual precautions, when the Superior was taken ill, the other fathers, having no suspicion the disorder could be the plague, communicated freely with him at the beginning of his sickness. When the certain marks of the plague were discovered, the fathers were much frightened, but all of them escaped the infection.

"While Larnica and Famagusta suffered severely from the plague, it was decreasing fast at Nicosia; in which city it was computed there had died near twenty thousand Turks, and between four or five thousand Greeks and Armenians,—a vast mortality, in proportion to its population.

"Towards the end of May, the plague was sensibly on the decrease at Larnica, as well as most parts of the island. It had almost ceased at Fermagusta, after having in a manner desolated the city; and, in the adjacent country, hardly left hands sufficient to gather in the harvest. In June, pestilential accidents continued to happen from time to time at Larnica; but the sick, in general, recovered. The heat of the weather was now considerably increased, though sometimes interrupted by cool showery days, on which persons sick in the plague were observed to suffer remarkably.

"Te Deum was sung by the French on the 3d of July. In the course of that month, all the Europeans came out from confinement; and the island was, at length, delivered from the plague, which, if the computation generally received was true, had destroyed seventy thousand of the inhabitants."

After this account, can we fail to doubt the necessity for, or efficacy of, quarantines, or the danger of "touching a book or a paper." The plague entered Linsol, though no more exposed, according to common opinion, than Larnica. It spread with great rapidity among the inhabitants, and among such as had occasion to resort thither; and, though these latter transported themselves and the supposed contagion to the mountains and neighbouring villages, and even to Larnica, yet they infected no one. When the plague arrived at Larnica, it spread without regarding any of the supposed means of infection, staid its own time, and ceased in its own way. The Europeans, who continued to walk about, do not appear to have suffered. In the Convent of Terra Santa, which was *shut up*, the Superior was taken ill; and, though he communicated freely with his brethren, infected no one.

Nor does it appear that the natives are indifferent to their own safety. Probably they only adopt a more rational means of preservation. Instead of *shutting up*, and avoiding contact with hot bread and with unwashed money, they desert a pestilential spot,—"abandon their half-desolate houses, and seek refuge in the country."

---

THE general apprehension entertained of a contagious fever, sporadic in different parts of England, particularly on the eastern side of the island; the late events at Nottingham and at Ipswich [*see page* 451 *of this Number*], induce us to transcribe part of the following paper.

It is certain, that ague is much less known than heretofore. It becomes us, therefore, to consider, whether fever in a new form is rising among us.

"*Some Observations concerning the Fever which prevailed at Cambridge during the Spring of* 1815. By J. HAVILAND, M.A. Licentiate in Medicine, Fellow of St. John's College, and Professor of Anatomy in the University of Cambridge.

"The winter of 1814 was mild, without any unusual degree of sickness; a few cases only of fever occurred, but they were not violent, and the patients recovered easily after a week or ten days' confinement. During the greater part of January 1815, I was absent from Cambridge; on my return at the end of that month, there was said to be a good deal of illness in the town, but still not enough to create alarm. In a week or two afterwards, a report prevailed that there was a bad fever in some of the colleges. About this time I was requested to visit a woman, who was a servant to one of those colleges which was supposed to be suffering the most. I saw her first on a Saturday, the fifth day of the disease, and she then complained of having been ill since the preceding Tuesday, but not to such a degree as to prevent her from following her usual employment, which she had now for the first time been obliged to give up. I could not learn that she had been exposed to any infection, nor could she at all account for her illness: the symptoms were, great prostration of strength, languor, and head-ache, loss of appetite, white tongue, frequent pulse, and rather full and costive bowels. She called my attention most particularly to the irritation and inconvenience she experienced from a prolapsus uteri, to which she had been long subject, but which by her account had been now increased by her efforts to overcome the costiveness.

My first object was to relieve the bowels by a purgative draught, and this was done effectually during the night. The next morning, the sixth day of her illness, I found her with an increase of febrile heat, the headache was aggravated with an appearance of approaching delirium; the other symptoms were nearly the same as on the preceding evening. On the next, or seventh day, the delirium was decisive, and of a very active kind; kind: she was continually endeavouring to get out of bed, and desirous of obtaining a knife to destroy herself, which she once attempted, and to prevent which two or three persons were constantly in attendance upon her: during the rest of her illness, she remained in the same state, almost without intermission, sometimes struggling violently, and almost always, when awake, talking loudly. On the following day, as this treatment had been ineffectual, I gave directions for a large blister to be applied to the head, and the whole body to be frequently sponged over with the same mixture of vinegar and water; this, at first, appeared to relieve her, by abating the heat of the skin, and was evidently grateful to her feelings, but it soon lost its effect, and I determined, on the tenth day of her illness, which was six days after I first saw her, on trying the cold affusion. Her pulse was then 120 in a minute, with active delirium; her skin was parched, and she complained of great heat and thirst: during the night before, she had escaped from her attendants, had run into the street, and after rolling herself in the kennel, returned of her own accord; when asked why she had done so, she said she had been looking for the river to cool herself. The affusion at first reduced her pulse from 120 beats to 108, she was more composed, and had some sleep. It was repeated several times during this and the following day, but the benefit arising from it gradually diminished. I saw her soon after it was last done, the morning of the twelfth day; her extremities were cold, her pulse was fluttering, and she appeared quite exhausted. I then thought it necessary to give some wine, but was soon obliged to discontinue it from the violent delirium it seemed to produce. The quantity she took at this time was inconsiderable, not altogether amounting to half a pint. The skin, however, did not recover its former morbid degree of heat. From this time till the day she died, which was the seventeenth of her illness, she was evidently getting worse, the pulse varied from 120 to 130 beats in the minute, the tongue was dry and brown, and the delirium unabated: during this period, and I had observed the same thing from the time of my first seeing her, though in a less degree, there was very considerable tremor and subsultus of the muscles, though the actual muscular debility, even to the last, was not so great as might have been expected. Within a very few hours of her death, I saw her standing unsupported in bed, whilst the attendants were shifting it, and she was then able to get out of bed with very trifling assistance. During the last thirty-six hours, she took wine freely. The bowels were constantly irregular, with a disposition to costiveness, but stools were procured at least once in twenty-four hours. I should mention, that, during the time the cold affusion was employed, little or no medicine was given internally, and that afterwards she took the camphor mixture with the compound spirit of ether. I have been thus particular in my account of this case, because it was the first I saw, and also the only fatal one. During my attendance on this woman, I was called to see a gentleman, who, together with general fever, had a violent and and acute pain in the right hypochondrium, with great tenderness, and a pulse full and strong. I bled him freely, at first from the arm, and afterwards by cupping and leeches; each of which gave considerable relief: the disease, however, went through its course of about three weeks, with many of its symptoms not unlike those which characterized the prevailing complaint; but, as there was some obscurity in the symptoms of this case, I shall forbear to relate it in detail.

"The next person, by whom I was consulted, was a student of that college in which the fever had been particularly fatal. The symptoms were nearly the same as those which I have described above, and the headache was not less strongly marked. As the cold affusion had afforded so little relief in the former case, after opening the bowels, and applying a blister to the nape of the neck, I determined, the third day of the disease, on trying the effect of leeches, three to each temple; an almost immediate alleviation of the pain and confusion of the head followed, with an abatement of the fever: the patient gradually became better, and in a little more than a week I discontinued my attendance.

"I was called to see another gentleman on the second day after the attack; and, after ordering an emetic, which operated also upon the bowels, the application of leeches to the temples had an equally good effect in preventing the headache, and removing the tendency to delirium, though the fever still went through its course, unattended by these particular symptoms. The remainder of my experience, in successive cases, began at a somewhat more advanced stage of the disease, and the whole may be generalised by the following observations.

"The attack was usually slow and insidious; for some days the patients walked about complaining of nothing but headache, and a deranged, which was commonly a costive, state of the bowels. The headache continued to increase, accompanied with great languor, until these symptoms compelled the sick to call for medical assistance, when I found the tongue to be white and thickly coated, and the pulse variable from 100 to 120; there was not always an increased heat of the surface, and in some cases, on the other hand, there was not merely a sensation of cold complained of by the patient himself, but it was sensible also to the feelings of others. I saw, indeed, no case in which there existed that dry and hot state of skin, which more immediately calls for the cold affusion, excepting that above related, and the success attending the practice in this instance was not such as to lead to its repetition. Cold affusion was accidentally tried in the case of a gentleman, who was recovering from the severity of his illness, and whom we had hoped to declare convalescent in a few days. He awoke early in the morning, hot, as he described it, but as I believe in a state of perspiration; and, fancying that the cold bath might be of service, persuaded his nurse, as a substitute for this, to throw cold water over his body: during the following day he relapsed, and suffered more from from the second than from the first attack; so that for some days his recovery was doubtful."

"Through the kindness of the medical gentlemen who attended, I had an opportunity of being present during the examination of the body of one gentleman, Mr. J. W., who had died of this disease, but whom I had not seen in the course of his illness. The examination took place twenty-three hours after death. The head was first opened, and in so doing the dura mater was cut through: there escaped a considerable quantity of a serous fluid, it was not easy to estimate the precise quantity, but it did not amount to less than four flui-drachms. The substance of the brain was particularly firm, and showed, on being cut through, an unusual degree of vascularity. The ventricles were distended with serum. The abdominal viscera were of a healthy appearance, except that the bowels were much distended with flatus.

"It became a very interesting question to determine whether or not the fever was infectious: at first, I was disposed to think it was not, for I could not hear of any facts that favoured an opposite conclusion: latterly I have doubted if this opinion was correct, and the following circumstance confirms these doubts: A servant girl, who was ill, was consequently sent home, from a family in the town, to her friends, who lived at a cottage distant about ten miles from Cambridge, at Stretham Ferry; and her disease proved to be the fever, of which she recovered; nearly all the family became afterwards ill of the same complaint, of which the father died. I have since heard of other cases of a similar nature. But the fever certainly did not strongly partake of this character, for servants, nurses, and medical attendants, who were particularly exposed to the disease, did not suffer from it more than others. In the two larger colleges, only one case occurred within the walls of each. The proportion of cases that died of this disease was certainly great. It would be extremely difficult to ascertain the exact number of persons that were ill, nor would it be more easy to find out, in the number of deaths that occurred in the town of Cambridge, how many arose from this cause. In the university it was generally understood that nine persons had died of fever; and, from all I can collect, it would appear that the number of decided cases was less than fifty. Many others were indisposed, but not to a degree to excite any alarm, and readily got well with the use only of some evacuant medicines.

"It is remarkable, that this fever confined its attacks, with few exceptions, to young persons. In the town, the children were the chief sufferers, and many died, especially amongst the poor. In the university, it was the younger part only that was ill; of those who died the oldest was about twenty-five, and the others were from nineteen to twenty-three years of age; I do not know a single member of the university above thirty years old who was ill of fever.

"As Cambridge has always, and with great justice, been considered as an extremely healthy town, I may be expected in this place place to say something about the cause of the late unhealthiness of the place: but, as this is a subject on which much difference of opinion exists, I shall avoid entering upon it for the present, and shall only observe, that I am disposed to attribute it, in great measure, to the state of the drains and ditches, which have been much neglected of late, whilst the population has greatly increased.* The cause, whatever it might be, was apparently local: for some time, the disease did not show itself in the neighbouring villages, and, when it did make its appearance, it was only in a partial manner; nor did those villages, whose situation is low, suffer more than others. I understood from a very respectable practitioner of Ely, which is situated only sixteen miles from Cambridge, and very much surrounded by fens, that that town and neighbourhood were remarkably free from fever, and that the only cases he had seen were in the family I have alluded to above, at Stretham Ferry."

We have omitted much concerning the treatment, our principal object being to ascertain the rise and progress of the fever.

---

### CRITICAL ANALYSIS

"* Active measures are now taking to remove this cause of complaint."

